# Hybrid-Transcriptome Sequencing and Associated Metabolite Analysis Reveal Putative Genes Involved in Flower Color Difference in Rose Mutants

**DOI:** 10.3390/plants8080267

**Published:** 2019-08-05

**Authors:** Ping Huang, Furong Lin, Bin Li, Yongqi Zheng

**Affiliations:** State Key Laboratory of Tree Genetics and Breeding, Laboratory of Forest Silviculture and Tree Cultivation, Research Institute of Forestry, Chinese Academy of Forestry, Beijing 100091, China

**Keywords:** *Rosa*, flower color variation, flavonoid biosynthesis, transcriptome, metabolite analysis

## Abstract

Gene mutation is a common phenomenon in nature that often leads to phenotype differences, such as the variations in flower color that frequently occur in roses. With the aim of revealing the genomic information and inner mechanisms, the differences in the levels of both transcription and secondary metabolism between a pair of natural rose mutants were investigated by using hybrid RNA-sequencing and metabolite analysis. Metabolite analysis showed that glycosylated derivatives of pelargonidin, e.g., pelargonidin 3,5 diglucoside and pelargonidin 3-glucoside, which were not detected in white flowers (*Rosa* ‘Whilte Mrago Koster’), constituted the major pigments in pink flowers. Conversely, the flavonol contents of petal, such as kaempferol-3-glucoside, quercetin 3-glucoside, and rutin, were higher in white flowers. Hybrid RNA-sequencing obtained a total of 107,280 full-length transcripts in rose petal which were annotated in major databases. Differentially expressed gene (DEG) analysis showed that the expression of genes involved in the flavonoid biosynthesis pathway was significantly different, e.g., *CHS*, *FLS*, *DFR*, *LDOX*, which was verified by qRT-PCR during flowering. Additionally, two *MYB* transcription factors were found and named *RmMYBAN2* and *RmMYBPA1,* and their expression patterns during flowering were also analyzed. These findings indicate that these genes may be involved in the flower color difference in the rose mutants, and competition between anthocyanin and flavonol biosynthesis is a primary cause of flower color variation, with its regulation reflected by transcriptional and secondary metabolite levels.

## 1. Introduction

Rose is one of the most important ornamental woody plants belonging to the genus *Rosa* of the Rosaceae family, and its original species and cultivar present diverse flowers, delicate fragrances, and ornamental traits. Roses have also been widely used as garden ornaments, cut-flowers, food, and medicine over thousands of years, and the rose has accounted for more than one-third of the total cut flower industry worldwide [1,2]. Over the past 5000 years, over 30,000 cultivated varieties have been selected and bred by the hybridization of certain original species and their cultivars, which are distributed across China, Europe, and the Middle East. Frequent interspecies crossbreeding between cultivated varieties and polyploid wild species has made the classification of roses very difficult, and these kinds of extensive hybridizations have led to the diversification of floral traits [3]. Floral traits have always attracted attention from breeders and scientists, and flower phenotypes result from the complex interaction of molecular and cellular processes with environmental factors [4]. Therefore, an understanding of the inner genetic regulation and control mechanisms is not only of interest to fundamental science, but is also an opportunity to enhance the knowledge regarding how these characteristics were selected or introduced to new cultivated varieties for improving the quality of rose [5]. Polyploidy, interspecies hybridizations, and a lack of effective genetic transformation systems have continued to be impeded to characterize gene function in roses. However, a large number of molecular markers have been developed in the last twenty years, e.g., simple sequence repeats (SSRs) and single nucleotide polymorphisms (SNPs) [2,6,7,8,9]. These markers have been used to identify different rose varieties, establish a DNA profiling database, construct a genetic linkage map, and genome-wide association analysis of flower traits [10,11,12].

Floral color is one of the most prominent traits in ornamental plants. In roses, petal color exhibits a wide range of variation in nature, except blue. Anthocyanidin, which is a kind of flavonoid found in plants, has been demonstrated to produce primary floral pigments: Pink or red petals result from anthocyanin and its derivatives, and yellow or orange petals may contain some carotenoid and anthocyanin [13]. The biosynthesis pathways of flavonoids have been well characterized in *Arabidopsis*, *Petunia*, and other species [14,15]. Flavonoids are derived from the phenylalanine metabolism pathway: A specific substrate is catalyzed by phenylalanine ammonia-lyase (PAL), chalcone synthase (CHS), chalcone isomerase (CHI), and flavanone 3-hydroxylase (F3H), and flows into anthocyanin biosynthesis by dihydroflavonol 4-reductase (DFR), anthocyanidin synthase (ANS) and UDP-glucose—flavonoid 3-*O*-glucosyltransferase (UFGT) or the biosynthesis enter into flavonol pathway by flavonol synthase (FLS). Co-expression and regulation of these related genes have been detected in some plants [16]. The composition of anthocyanidin and flavonol results in the diverse phenotype of the flower, from light to dark, or monochrome to polychrome [17]. Additionally, the co-pigment, intracellular pH, and external environment also play significant roles in floral color phenotypes [18,19,20]. The recent publications of several high-quality genomes of *Rosa* species will promote the development of fundamental research on *Rosa* spp. [21,22,23]. Nevertheless, the molecular mechanisms that control floral color may vary among different roses, because modern roses represent a complicated cultivated population [24,25].

Flower traits vary frequently in roses, e.g., floral color and type, and that is why many of the rose varieties that have been selected are based on natural mutations. These varieties are valuable research materials for the comparison and analysis of plant trait variations because they are nearly isogenic when compared to each other. With the rapid development of biotechnology, new approaches and tools could be used to reveal the internal mechanisms of phenotypic expression in plants, especially those involved in secondary metabolite biosynthesis [26,27,28,29,30]. Transcriptome analysis has been popular for studying differences in gene expression in the context of floral development and transition, and in response to stress and exogenous hormones; therefore, vast and useful transcript information has been found in rose using next-generation sequencing (NGS) [31,32,33,34]. Moreover, long-read sequencing is one of the most important merits of the PacBio platform, which can provide 3–10 kb of information from DNA or RNA, and will contribute to gene isoform reconstruction, novel isoform, and fusion gene discovery. Additionally, the annotation will be more accurate than NGS [35,36,37]. Combining their strengths, hybrid-seq strategy (PacBio + NGS) will consider both read lengths and sequencing accuracy in transcriptome analysis, and reduce the appearance of false assembly [38].

In this study, we attempt to understand the difference in transcriptional and secondary metabolism levels between two rose varieties with different phenotypes, using both hybrid-transcriptome sequencing and metabolite analysis on two rose varieties with different flower colors: *Rosa* ‘Margo Koster’ (pink) and *Rosa* ‘White Margo Koster’ (white). For RNA-seq, we constructed and sequenced cDNA libraries of petals at the full-bloom stage using the Illumina HiSeq 2500 and PacBio RSII platforms. In addition, relative quantification of flavonoid and anthocyanin in the petals was analyzed by high-performance liquid chromatography–diode array detection–electrospray ionization with high-resolution mass spectrometry (HPLC–DAD–ESI–HR–MS). Candidate genes related to pigment biosynthesis in the petals were verified by qRT-PCR at different developmental stages.

## 2. Results

### 2.1. Survey on Hybrid RNA Sequencing

**Figure 1:**
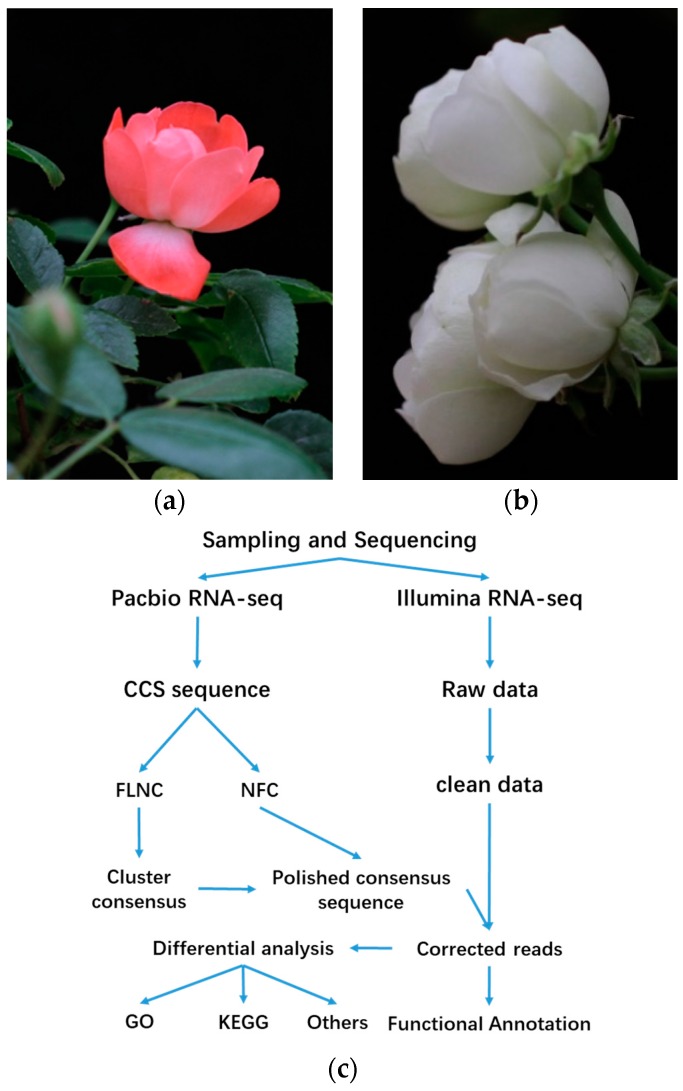
Petal material of *Rosa* ‘Margo Koster’ (**a**) and *Rosa* ‘White Margo Koster’ (**b**) at blooming stage used for RNA-seq (**c**) work process of bioinformatics analysis of hybrid RNA sequencing in this study.

Samples from rose petals at the blooming stage were used for hybrid RNA sequencing (Figure 1). The results show that 538.39 million clean sub-reads were obtained using PacBio sequencing technology (Table 1), and 156.96 million clean reads and 156.99 clean reads for *Rosa* ‘Margo Koster’ and *Rosa* ‘White Margo Koster’ were obtained on the Illumina platform, respectively (Table 2). Clean data have been uploaded to the National Center for Biotechnology Information Sequence Read Archive (accession number: SRP132317).

**Figure 2:**
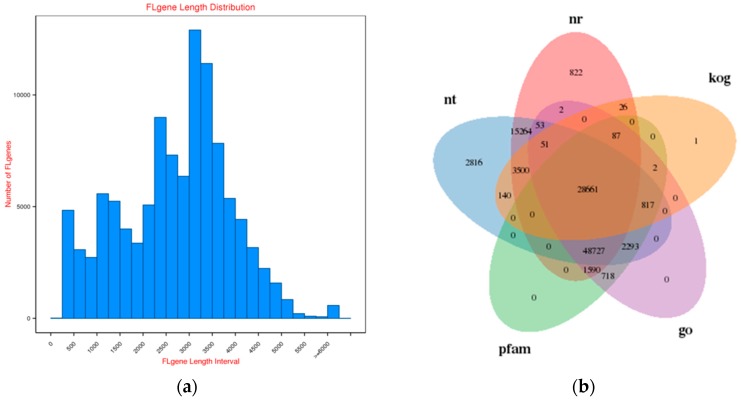
Full-length gene from SMRT sequencing and their annotation in main database. (**a**) The length distribution of full-length gene. Horizontal axis indicates the different length interval of full-length; vertical coordinates indicate the number of full-length transcripts. (**b**) Gene annotation of full-length gene against main databases, including Nucleotide Sequence Database, Nt; Non-Redundant Protein Sequence Database, Nr; Protein families and domain database, Pfam, Gene Ontology, GO, and euKaryotic Orthologous Groups database, KOG.

A total of 463,753 circular consensus sequences (CCS) were generated from three single-molecule real-time sequencing (SMRT) cells in a non-normalized bin (1–2, 2–3, and 3–6 kb), including 365,407 (78%) full-length non-chimeric (Flnc) reads and 100,346 (22%) non-Flnc reads. The average length of an Flnc read was 2591 bp. To enhance the quality and accuracy of SMRT transcripts and quantify gene expression, a total of 313 million reads from two rose varieties were generated by the HiSeq 2500 platform, and over 83% of these reads were mapped to the reference transcripts generated by PacBio RSII. Raw SMRT sequencing reads were processed by SMRTLinkv4.0, and all SMRT sub-reads were corrected using over 300 million HiSeq reads as input data by Proovread. Then, all SMRT sub-reads were processed by CD-HIT-EST to remove the redundant sequences. Finally, a total of 107,280 transcripts were generated with an N50 of 3267 bp and an average length of 2710 bp. The transcripts longer than approximately 1 kb accounted for more than 90%; their length distribution is illustrated in Figure 2a.

### 2.2. Functional Annotation of Rose Variety

Annotation of the transcripts was carried out to generate a hybrid-transcriptome database of rose variety. The results of gene function annotation showed that a total of 105,582 (98.41%) transcripts were annotated in at least one database, and a total of 21,857 transcripts were annotated in seven databases, including Nucleotide Sequence Database, Nt; Non-Redundant Protein Sequence Database, Nr; Protein families database, Pfam, Gene Ontology, GO, and euKaryotic Orthologous Groups database, KOG (Figure 2b), and the detailed annotation information is shown in Supplemental File 1. Additionally, more than 3000 potential transcription factors (TFs) were predicted by iTAK (Supplemental Figure S1). As a result, a total of 83,001 transcripts were mapped to GO terms. Assignments were given to biological processes (29.33%), molecular functions (47.53%), and cellular components (23.14%) (Supplemental Figure S2). Among all of the GO terms, the vast majority were related to cell components (148,617), biological process (240,852), and molecular functions (117,253). In addition, all of the transcripts were mapped to records in the KOG database, and KOG annotations were retrieved with a total of 33,285 putative proteins that were functionally classified into at least 25 protein families (Supplemental Figure S3). Finally, KEGG (Kyoto Encyclopedia of Genes and Genomes) pathway analysis was performed to assign biological pathways to all of the transcripts; the results showed that 43,005 transcripts were assigned to 128 KEGG pathways and clustered into 19 groups, e.g., transport and catabolism, membrane transport, folding, sorting, and degradation (Supplemental Figure S4).

### 2.3. Simple Sequence Repeat (SSR) Detection

Simple sequence repeat (SSR) is a co-dominant, abundant molecular marker in the genome which has been used for fundamental plant science research, such as population genetics, QTL mapping, and DNA profiling. In total, 40,074 putative SSR motifs (di-hexanucleotides) were identified in 45,782 full-length transcripts, in addition to 56,828 mononucleotide motifs. Out of these full-length transcripts, a total of 20,252 (20.89%) included more than one SSR, whereas a total of 28,391 (29.29%) was presented in compound formation. The highest frequency of SSR was of mononucleotide (58.64%), followed by dinucleotide (26.06%) and trinucleotide (14.24%), as shown in Table 3. Compared with next-generation sequencing, the efficiency of mining putative SSR motifs and their compound formation was improved greatly using the PacBio technique, owing to its ability to obtain long reads. The primers information of candidate SSR markers is listed in Supplemental File 3. These putative SSR loci may be potential candidates for the development of genic SSR markers and be informative in constructing genetic maps or describing genetic variation in roses.

### 2.4. Analysis of Differentially Expressed Genes (DEGs) Identified in the Two Libraries

**Figure 3:**
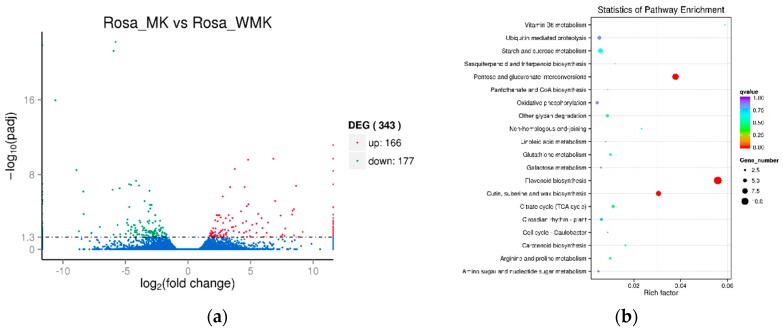
Differentially expression genes (**a**) and their KEGG enrichment (**b**) in the petal of two rose varieties. *Rosa* ’MK’ and *Rosa* ’WMK’ indicate *Rosa* ‘Margo Koster’ and *Rosa* ‘White Margo Koster’.

Analysis of differentially expressed genes (DEGs) was performed between *Rosa* ‘Margo Koster’ (pink) and *Rosa* ‘White Margo Koster’ (white) based on the FPKM (Fragments Per Kilobase Million) with the thresholds P_adj_ < 0.05 and fold change >2. The results show that there were 343 DEGs (166-up- and 177-down-regulated) between two rose varieties (Figure 3, Supplemental Table S1, and Supplemental File S2). A total of 125 up-regulated DEGs and 138 down-regulated DEGs were enriched into GO terms. The main terms were “catalytic activity” including 178 DEGs. A total of 125 up-regulated DEGs and 138 down-regulated DEGs were enriched into KEGG pathways, the main pathways of DEGs enrichment were “Flavonoid biosynthesis” (ko00941), “Pentose and glucuronate interconversions” (ko00040) and “Cutin, suberine and wax biosynthesis” (ko00073) (Figure 3).

### 2.5. Characterization and Expression Analysis of Genes Involved in Flavonoids and Associated Biosynthesis Pathway

Pigments in rose flower are mainly flavonoids, anthocyanins, and carotenoids, and the different compositions of these secondary metabolites contribute to the diversity of flower color [39]. According to gene annotation and the classification of KEGG, about 6% of transcripts were assigned to secondary metabolite biosynthesis, e.g., a total of 350 transcripts were related to the phenylpropanoid biosynthesis, and a total of 197 transcripts coded 13 enzymes which catalyzed the synthesis of various flavonoids, including chalcone, flavone, flavonol, and their derivatives (Supplemental Table S2). Hence, genes in the flavonoid, anthocyanin, and flavonol biosynthesis pathways were the focus of this study. Based on the gene annotation and DEGs analysis, the targeted gene involved in flavonoids and the associated biosynthesis pathway were screened and compared. The results showed that the expression of genes encoding chalcone synthase (CHS), flavonol synthase (FLS), dihydroflavonol-4-reductase (DFR), leucoanthocyanidin dioxygenase (LDOX), and glucosyltransferase (GT) significantly differed between the two rose varieties (Table 4). Some genes in the flavonoid pathway had differential expression levels in *Rosa* ‘Margo Koster’ compared to *Rosa* ‘White Margo Koster’, suggesting that these changes affect the anthocyanin and flavonol biosynthesis pathways, respectively, due to regulation at the transcriptional level.

### 2.6. Expression Profiling of Flavonoid Biosynthesis Related Genes During Flowering

**Figure 4:**
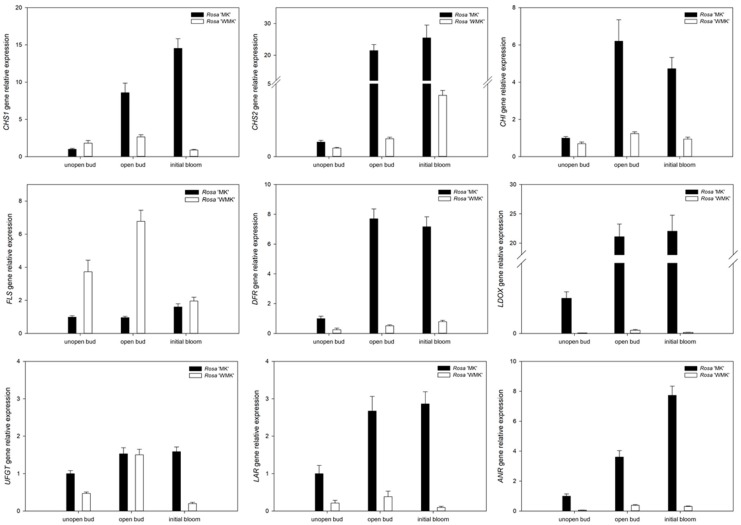
Graphs showing gene expression in the anthocyanin biosynthesis pathway. Key enzymes and their differential gene expression during flowering stages (unopened bud, opened bud, and initial bloom) were analyzed by qRT-PCR. The expression level of each gene is relative to that of *ACTIN* reference in *Rosa* ‘Margo Koster’ at unopen bud stage. Each data point is the average of three biological repeats. Error bars indicate standard deviations (SDs). CHS, chalcone synthase; CHI, chalcone isomerase; FLS, flavonol synthase; DFR, dihydroflavonol-4-reductase; LDOX, leucoanthocyanidin dioxygenase; UFGT, anthocyanidin-3-*O*-glucosyltransferase; ANR, anthocyanidin reductase; LAR, leucoanthocyanidin reductase.

Anthocyanin is well known for its role in the coloration of flowers, and flavonol is also an important co-pigment in plants. A previous study has reported that various flower colors in plants can be attributed to differences in the biosynthesis pathways or accumulation of anthocyanin [40]. Thus, our results also support the findings of previous research. To verify the results of transcriptome sequencing and investigate the patterns of gene expression in petals during flowering, the expression of genes related to the aforementioned biosynthesis pathways were analyzed in petals by qRT-PCR during different flowering stages, including unopen bud, open bud, and initial bloom. Firstly, PCR efficiency of each mRNA and Raw Cq data of qRT-PCR was calculated and is shown in Supplemental Table S3 and Supplemental Figure S5, and the relative gene expression level is shown in Figure 4. These results show that the expressions of *CHS* and *CHI* in *Rosa* ‘White Margo Koster’ were lower than that in *Rosa* ‘Margo Koster’, and expressions of *DFR* and *LDOX* were almost undetectable in white flowers during flowering. These genes are known to contribute to pigment biosynthesis and accumulation. Further, *LAR* and *ANR*, which, respectively, code enzymes for catalyzing the transformation of leucocyanidin and anthocyanin into procyanidin, also had lower expression levels in white flowers during flowering, indicating less accumulation of anthocyanin or its precursor. By contrast, the expression of *FLS* in the petals of *Rosa* ‘White Margo Koster’ was higher than that in *Rosa* ‘Margo Koster’ during flowering (Figure 4).

### 2.7. Major Classes of Flavonoids and Anthocyanins in the Two Rose Varieties

**Figure 5:**
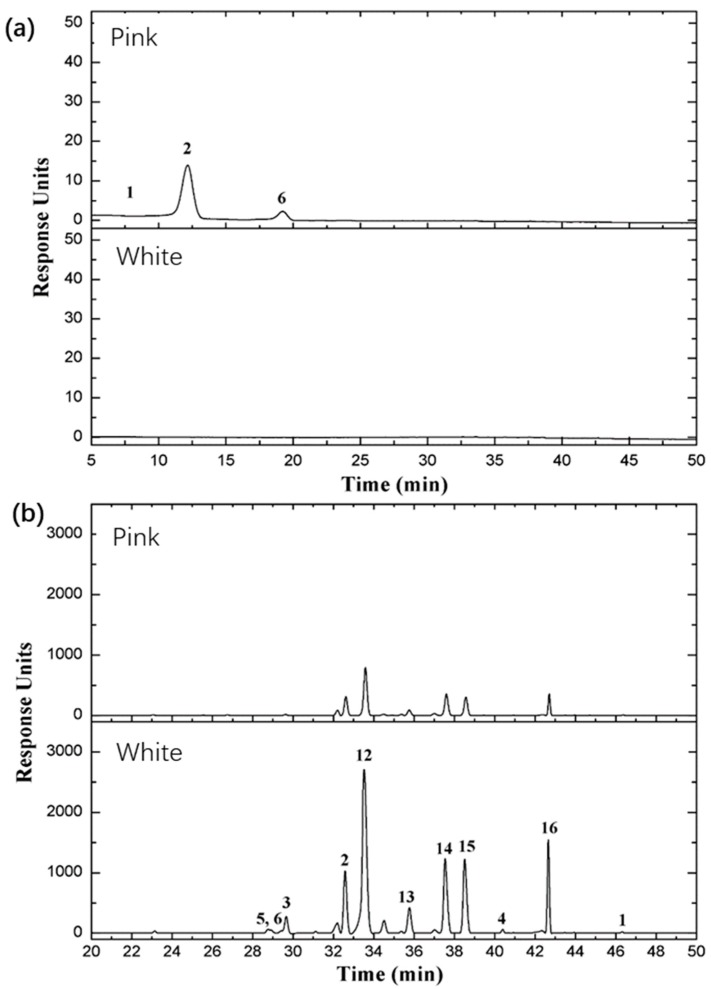
Comparison of (**a**) anthocyanin, and (**b**) flavonoid composition between *Rosa* ‘Margo Koster’ (pink) and *Rosa* ‘White Margo Koster’. The horizontal axis represents retention time, the vertical axis represents response units, and numbers on the peaks represent different compositions of anthocyanin and flavonoid. (**a**) 1-cyanidin 3,5-diglucoside, 2-pelargonidin 3,5-diglucoside, 6-pelargonidin 3-glucoside; (**b**) 2-kaempfeol-3-rutinoside, 12-kaempferol-3-glucoside, 13-kaempferol-3-*O*-α-d-arabinoside, 14-kaempferol-3-*O*-β-glucopyranosyl-7-*O*-α-rhamnopyranoside, 15-kaempferol-3-rhamnopyranoside (afzelin), and 16-multiflorin A.

As transcription levels revealed, the biosynthesis and accumulation of flavonoids and anthocyanins are different between the two rose varieties. To discuss the correlation between transcription and metabolites, we examined the accumulation of flavonoids, anthocyanins, and procyanidin in rose petals in this study. The results showed that five anthocyanin derivatives and two procyanidins were detected and identified, including pelargonidin, cyanidin, peonidin, procyanidin B1, and proanthocyanidin, and 16 flavonoids were detected and identified, including kaempferol, quercetin, rutin, hyperoside, (−)-epicatechin, (+/−)-catechin hydrate, and multiflorin A. The differential accumulation of flavonoids and anthocyanins in the petals of two rose varieties is illustrated in Figure 5, and the relative abundance of these metabolites is shown in Table 4. These findings indicate that the rose varieties exhibited a wide variation of pigments in their petals, including cyanidin, peonidin, and pelargonidin, as well as other pigments; pelargonidin 3,5 diglucoside and pelargonidin 3-glucoside were the main pigments in the petal of *Rosa* ‘Margo Koster’, and a small quantity of cyanidin 3,5-diglucoside and peonidin 3,5-diglucoside were detected in *Rosa* ‘Margo Koster’ (Table 5, Figure 5). By contrast, neither anthocyanins nor their derivatives were detected in the petal of *Rosa* ‘White Margo Koster’, but the relative flavonol content was higher in white petals than in pink petals, and their glycosylated derivatives included kaempferol-3-glucoside, kaempferol-3-rhamnopyranoside, and quercetin-3-glucoside. These results suggest that the accumulation of anthocyanins is the main cause of the different phenotypes observed in the two studied rose varieties and corresponds with the observed transcriptional variation. The results also confirm that the accumulation pattern of secondary metabolites mostly correlates with the transcription level of candidate genes in the biosynthesis pathway.

### 2.8. Alignment of Deduced Amino Acid Sequences of Two R2R3-MYB TFs

**Figure 6:**
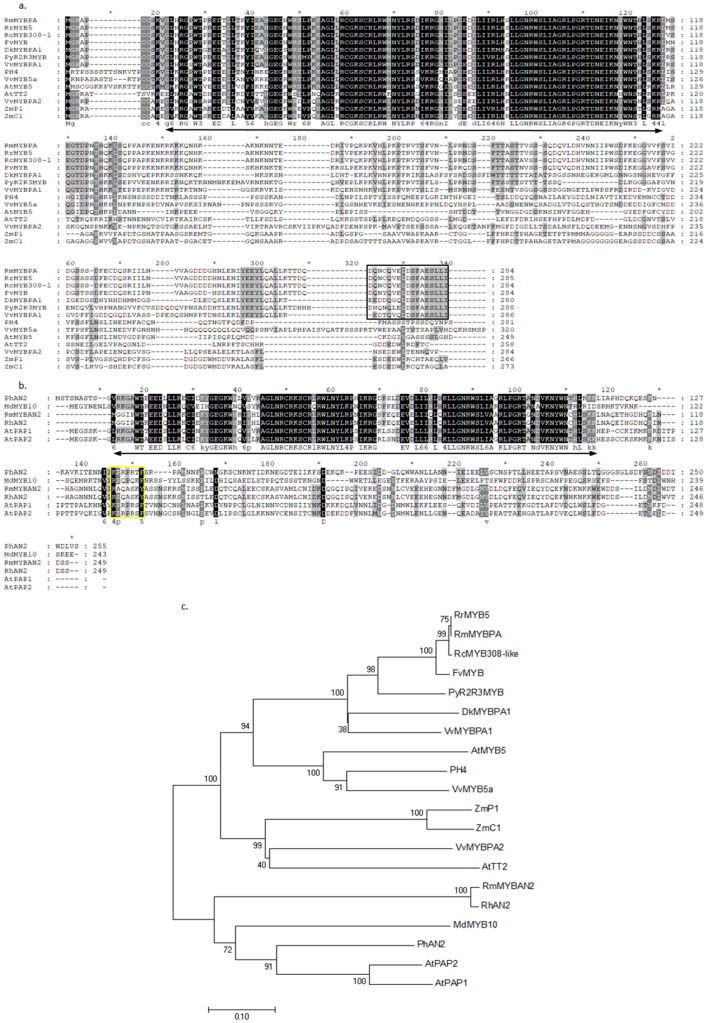
Analysis of deduced amino acid sequences of two R2R3-MYB TFs. (**a**) Alignment of deduced amino acid sequences of the AN2 subgroup of MYB TFs, and (**b**) alignment of deduced amino acid sequences of the TT2 and PA specific-MYB regulators. ZmC1, ZmPl (maize), AtTT2, AtMYB5 (*Arabidopsis thaliana*), PH4 (*Pharbitis hybrid*), VvMYB5a, VvMYBPA1, VvMYBPA2 (*Vitis vinifera*), PyR2R3MYB (*Prunus yedoensis*), DkMYBPA1 (*Diospyros kaki*), RrMYB5 (*Rosa rugusa*), RhMYB308-like (*Rosa chinensis*), and FvMYB (*Fragaria vesca*). The R2R3MYB domains are indicated by black arrow, yellow box indicates a C-terminal conserved motif [K/R]Px3[K/T][F/Y] in AN2 subgroup of R2R3MYB. Black box indicates a C-terminal conserved motif in the PA-specific MYB regulators. (**c**) A neighbor-joining phylogenetic tree of plant R2R3MYB sequences. The scale bar represents 0.1 substitutions per site and the numbers next to the nodes are bootstrap values from 1000 replicates. The deduced amino acid sequences were retrieved from the DDBJ/EMBL/GeneBank databases, and the accession numbers are as follows AtPAP1(NP_176057.1), AtPAP2(NP_176813.1), RhAN2(BAM36703.1), PhAN2(AAF66727.1), MdMYB10(AAF66727.1), and ZmC1(sp|P10290.1).

Members of the MYB protein superfamily contain one or several conserved MYB DNA-binding domains. In plants, the predominant family of MYB proteins is the R2R3-type, which is characterized by two repeat MYB domains, and previous reports have indicated that R2R3-MYB genes are involved in many aspects of secondary metabolism, as well as the identity and fate of cells in plants [42,43]. A total of 339 transcripts were predicted as MYB TFs by iTAK. In the DEGs, we found that two full-length transcripts named *RmMYBAN2* and *RmMYBPA*, which encoded 249 and 284 amino acids, respectively, showed different expression levels in the flowers of two rose varieties. Alignments of amino acid sequences showed that the region of R2R3 repeats was highly conserved in plants, but that the downstream region was divergent regarding sequence and length. A part of this motif, [K/R]Px_3_[K/T][F/Y], which was found in the RmMYBAN2, is thought to be a conserved in the AN2 subgroup, according to a previous report [44]. Based on full-length amino acid sequences, a phylogenetic tree is illustrated in Figure 6c. These results indicate that RmMYBAN2 clustered to an AN2 subgroup, and RmMYBPA clustered to another group which is involved in regulating the biosynthesis of procyanidin, including TT2-like protein and PA-specific regulator [45]. In the RmMYBPA sequence, we cannot find the short motif KAx[K/R]C[S/T], which was conserved in the C1 subgroup [46]. These results indicate that RmMYBPA does not belong to the C1 subgroup. However, the alignment results showed that the RmMYBPA protein has the typical features of plant MYB TFs, and its C-terminal protein also had a high similarity to PA-related regulators [47].

### 2.9. Expression Patterns of Two R2R3-MYB TFs During Flowering

**Figure 7:**
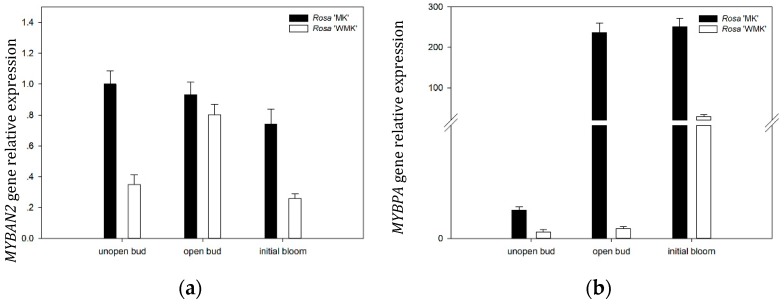
Gene expression level of (**a**) *RmMYBAN2* and (**b**) *RmMYBPA* of the *Rosa* ‘Margo Koster’ and *Rosa* ‘White Margo Koster’ during flowering (unopened bud, opened bud, and initial bloom). The expression level of each gene is relative to that of *ACTIN* reference in *Rosa* ‘Margo Koster’ at unopen bud stage. Each data point is the average of three biological repeats. Error bars indicate SDs.

To understand the gene expression pattern of *RmMYBAN2* and *RmMYBPA* during flowering in two rose varieties, we investigated the transcription levels of *RmMYBAN2* and *RmMYBPA* at three developmental stages by qRT-PCR, and *RhACTIN* was chosen for the normalization of gene expression. The results showed that the gene expression level of *RmMYBAN2* in *Rosa* ‘Margo Koster’ was higher than that in *Rosa* ‘White Margo Koster’ at unopen bud and initial bloom stage (Figure 7a). For the *RmMYBPA*, its expression level in *Rosa* ‘Margo Koster’ rapidly increased and remained stable after unopen buds stage, and subsequently, its expression levels in *Rosa* ‘White Margo Koster’ were always low and remained stable before the initial bloom, and the expression levels of *Rosa* ‘Margo Koster’ during flowering were higher than *Rosa* ‘Margo Koster’ (Figure 7b).

## 3. Discussion

### 3.1. Hybrid Sequencing Strategy Provides More, Better-Quality Information

New sequencing techniques have emerged during the last 10 years when NGS and SMRT were commercialized in different platforms. The hybrid strategy of RNA sequencing has previously been applied to study the secondary metabolite pathway in medicinal plants [28,48,49]. As we know, RNA sequencing using NGS has been extensively applied to rose studies [1,2,31,34], and in these reports, the average length of transcripts in assembled reads is no more than approximately 1.5 kb. In this study, the average length of transcripts was over 2.5 kb, which was much higher than the average length in previous reports using NGS sequencing. Longer transcripts can provide complete coding sequences of functional genes and contribute to improving the accuracy of gene functional annotation. Over 98% of transcripts have been annotated in the main database, which benefits from long reads. Nevertheless, the combination of sequencing approaches of both sets of data—correcting the SMRT reads using NGS reads—was used to obtain high-quality full-length transcripts of flower tissue in the opened stage. Additionally, SSR markers are also important tools for population genetic analysis and the construction of linkage mapping in roses [10,11,12], but in plants, the development of SSR markers in roses is neither highly efficient nor cheap. Recently, over 12,000 genomic and 2000 EST–SSR markers have been developed and identified by NGS technology in *Rosa* hybrids [2]. In addition to mononucleotides, over 40,000 candidate SSR sites, which are a great supplement for rose molecular markers, were found in our transcript pool. These candidate sites are informative and will be useful for fundamental research in roses, although they still need to be identified through classical PCR experiments or further bioinformatics analysis. However, this also indicates that the discovery efficiency of molecular markers using hybrid sequencing is also high in roses.

### 3.2. Differential Expression of Genes in the Petal of Two Rose Varieties

The pathways controlling petal color are well characterized in angiosperm plants [16], and the flower pigment in roses is reviewed [5]. Thus, gene modification has been demonstrated to be an effective way to change the flower color in rose; however, this trait is also affected by other environmental factors, such as vacuolar pH or co-pigments [50,51]. Regulation of metabolite flux towards a specific pathway is also an interesting and challenging problem in rose because of the wide genetic variation. It should also be taken into consideration that both of the studied varieties are almost isogenic compared to each other. Most DEGs in the pigment biosynthetic pathway are the consequence of, or reason for, phenotype variations. Hence, the DEGs of the flavonoid and anthocyanin pathway were focused upon in this study, e.g., the expression levels of *CHS*, *CHI*, *DFR*, and *LDOX* in pink flowers were significantly higher than in white flowers; these genes are correlated with the accumulation of anthocyanin in model plant species. CHS is the first rate-limiting enzyme which catalyzes to synthesized tetrahydroxy-chalcone, in the flavonoid biosynthesis. Lower expression of *CHS* will inhabit anthocyanin biosynthesis and result in a change of plant tissues [16,17]. Dihydroflavonol 4-reductase is considered a critical enzyme that functions later during anthocyanin biosynthesis [52,53]. It reduces dihydroflavonols to leucoanthocyanidins, and promotes the flavonoid to anthocyanin pathway, and then, ANS will convert colorless leucoanthocyanidins to colored anthocyanin, resulting in pigment accumulation. The expression level of these genes in our study indicates that the flavonoid can convert to colored anthocyanin in pink flower rather than white flower, owing to the high expression level of *DFR*, *ANS,* and other genes in the downstream pathway. Flavonols are a class of flavonoids which are important in plant development and UV defense, and their biosynthetic pathway is one of the branches of the flavonoid biosynthetic pathway. Flavonol synthase is an enzyme that catalyzes the conversion of dihydroflavonol to flavonol [54], resulting in the biosynthesis of colorless flavonol. In the white flower, a greater flow of flavonoids into the flavonol biosynthesis pathway is observed and the expression level of *FLS* is also higher than that of the pink flower. It might be related to the accumulation substrate. The accumulation of flavonol may be affected by the transcription regulator or other associated genes in the flavonol pathway [55,56,57,58]; however, the expression levels of flavonol-related regulators showed no difference between the two rose varieties.

Additionally, an MYB regulator named *RmMYBAN2* was found, which belongs to the R2R3-MYB gene superfamily involved in the regulation of anthocyanin biosynthesis [59]. Further analysis revealed that RmMYBAN2 belongs to the AN2 subgroup, which contains a conserved motif [K/R]Px_3_[K/T][F/Y], and this type of anthocyanin-related MYB TF is not divergent in reported dicot species [60]. It always co-infiltrated and interacted with TRANSPARENT TESTA, and activated the promoters of *DFR* and *ANS* in tobacco [61]. In this study, similar expression patterns of related genes were observed in pink rose during flowering. Then, another MYB TF named *RmMYBPA*, which is also a typical R2R3-MYB TF, but *RmMYBPA* does not belong to AN2 subgroup or C1 group. The result of homologous sequence analysis indicates that it may be orthologous, but a little divergent from the TT2-type, which participates in PA biosynthesis by activating the ANR and LAR genes in *Arabidopsis*, grape, and persimmon [62,63,64,65,66,67,68,69]. RmMYBPA protein is more similar to VvMYBPA1 and DkMYBPA1, these MYB TFs also control expression of the PA biosynthesis genes, including ANR and LAR, but it does not contain a conserved C-terminal motif [47,68].

### 3.3. Differential Accumulation of Flavonoid in Two Rose Varieties

**Figure 8:**
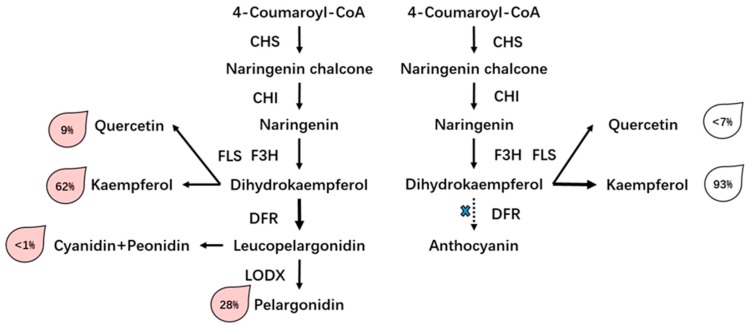
A putative model of the biosynthesis process of pigment accumulation in two rose varieties. The expression of *DFR* is switched off, and the substrate used for anthocyanin synthesis is shunted to flavonol synthesis; hence, *FLS* is up-regulated in the white flowers. The total output of flavonoids and anthocyanins was considered to be 100% and was used to evaluate the relative level of each product.

Flowering plants show a wide range of variation in their floral, foliage, and fruit colors because of an abundance of genetic variation and diverse environments. Previous reports show that this is due to the flexible flux of the flavonoid pathway and diverse regulation mechanisms, and the accumulation and composition of anthocyanin determine the scheme and density of flower color [70]. Previous reports have shown that pelargonidin is responsible for the orange to red color [15,71]. In this study, the findings indicate that pelargonidin and its derivatives are major pigments in pink flowers. Conversely, the main flavonoid in white flowers is flavonol, which is important in the response to abiotic stresses in plants [72]. However, the type of flavonol hardly differentiates between the two rose varieties, and we also found that flavonols were present in pink rose, and the gene expression level of *FLS* was also kept stable during flowering; hence, its content should be stable as one of the co-pigments in flowers. Thus, the relative quantitative content of flavonols, e.g., quercetin-3-glucoside and kaempferol-3-glucoside, was higher in white flowers than in pink flowers. These results also suggest that the majority of flavonoids tended to be converted in the flavonol pathway in white flowers, this kind of imbalance distribution of the flavonoid pathway has been observed in core eudicot plants [73]. We conclude that the phenotype of white flower results in a lack of anthocyanin accumulation, which may result from the low expression of related genes, such as *DFR* and *LDOX*, and subsequently, most flavones are converted to flavonols by *FLS* (Figure 8).

We report profiling analysis of a hybrid RNA-seq and metabolites to investigate the different flower color of rose varieties and the associated expression patterns of flavonoid biosynthetic genes in pink and white roses. The results of these investigations showed a set of putative genes involved in the flavonoid biosynthesis pathway has been found, and the imbalance or competitive expression of *DFR* and *FLS* genes might determine the formation of flower color in the two rose varieties. In addition, two complete *R2R3-MYB* TFs were discovered and their sequences were analyzed and classified; however, their biological functions require more experimental proof.

## 4. Materials and Methods

### 4.1. Plant Material

*Rosa* ‘Margo Koster’ is a kind of polyantha rose discovered by D. A. Koster (The Netherlands, 1935), and this cultivar originates from *Rosa multiflora*. *Rosa* ‘White Margo Koster’ is a somatic variant of *Rosa* ‘Margo Koster’, and its flower color was reversed from pink to white. *Rosa* ‘White Margo Koster’ was discovered by Viktor Teschendorff (Germany, 1939). Breeding records were obtained from a public website (www.helpmefind.com), and previous results of DNA profiling using SSR markers showed that these two varieties of rose have the same DNA fingerprints. Pot plants were grown in the greenhouse at the Chinese Academy of Forestry, Beijing, China. Petals of two varieties were collected at the blooming flower stage. Three biological repetitions of each variety were used for RNA sequencing on the NGS platform, and a mixture of petals from two varieties was used for RNA sequencing by the PacBio platform. Then, a mixture of six flowers from each variety was used for secondary metabolite analysis. Finally, petals of two varieties at three developmental stages—unopened bud, opened bud, and blooming flower—were used to verify gene expression differences via qRT-PCR. All the samples were immediately frozen in liquid nitrogen and then stored at −80 °C.

### 4.2. Identification and Quantification of Flavonoids and Anthocyanins in the Rose Petals

The procedures used to identify and quantify the anthocyanin and flavonoid compositions in the petals of both rose varieties were modified from a previously described method [74,75]. Briefly, 10 mg of lyophilized sample powder from each rose variety was extracted by 1 mL of a 0.1% acetic acid/methanol solution at 4 °C overnight. The samples were subsequently centrifuged for 10 min at 10,000 rpm, after which the supernatants were collected with a vacuum centrifuge concentrator (CV100-DNA, Aijimu, Beijing, China) for drying. The experimental materials were then stored at −20 °C. The dried extracts were redissolved in MeOH immediately prior to analysis, after which they were examined by a liquid chromatography quadrupole time-of-flight mass spectrometer (Q-TOF-MS, 6520 series classic G6520B) with a 1200 series HPLC instrument (Agilent Technologies, Palo Alto, CA, USA) as an electrospray ionization (ESI) source. The separation was carried out at 40 °C with a Thermo Scientific RP-C18 column (150 mm × 4.6 mm) that had a particle size of 55 µm. The gradient, which involved 0.1% formic acid (A) and acetonitrile (B) as the mobile phases, consisted of 0–4 min (5% B), 4–35 min (5–20% B), 35–55 min (20–50% B), 55–60 min (50–75% B), 60–62 min (100% B), 62–67 min (100% B), 67–68 min (5% B), and 68–73 min (5% B) segments. Operating conditions were as follows: A flow rate of 1.0 mL·min^−1^, positive ESI mode, capillary voltage of 3.0 kV, and 16 L·h^−1^ of nebulization nitrogen flow.

The compounds in the sample extracts were identified by comparison with the retention times of standards. Characteristics of the UV–Vis spectra of peaks and the mass spectrometric information were analyzed with MassHunter Qualitative software (Agilent, USA). The relative content of anthocyanins and flavonoids was calculated from the peak areas of the samples based on the intensity of the corresponding standard compounds, including kaempferol, quercetin, rutin, quercetin-3-glucoside, kaempferol-3-rutinoside, cyanidin, pelargonidin, cyanidin-3-rutinoside, pelargonidin-3,5-diglucoside, and proanthocyanidin B1, as well as proanthocyanidin. For compounds lacking corresponding standards, quantification was performed using similar compounds.

### 4.3. RNA Extraction, NGS Library Construction, and Sequencing

Total RNA was extracted using an mRNA Isolation Kit in accordance with the manufacturer’s protocol. RNA integrity was evaluated using an Agilent 2100 Bioanalyzer (Agilent Technologies, Santa Clara, CA, USA). The samples whose RNA integrity number (RIN) ≥7 were subjected to subsequent analyses. The libraries were constructed using a TruSeq Stranded mRNA LT Sample Prep Kit (Illumina, San Diego, CA, USA) in accordance with the manufacturer’s instructions. Double-stranded RNA-complementary DNA (cDNA) was synthesized using a SuperScript II Double-Stranded cDNA Synthesis Kit (part#18064014, Invitrogen, CA, USA) and random hexamer primers. The cDNA fragments were subjected to purification, end repair, and ligation to sequencing adapters. The ligation products were purified with magnetic beads and separated by agarose gel electrophoresis. A range of cDNA fragments (200  ±  25 bp) were excised from the gel and selected for PCR amplification as templates. These libraries were then sequenced on an Illumina sequencing platform (HiSeq2500), and 125 bp paired-end reads were generated.

### 4.4. Library Preparation and PacBio RSII Sequencing

The cDNA sample from mixed petal comprising both varieties was constructed for use in building a SMRT sequencing library. Three different libraries, each consisting of a cDNA insert size of <2 kb, 2–3 kb, and 3–6 kb, were prepared in accordance with the SMRT sequencing protocol using a Clontech SMARTer PCR cDNA Synthesis Kit and the BluePippin Size Selection System protocol as described by Pacific Biosciences (PN 100-092-800-03). These libraries were then sequenced on a PacBio RSII sequencing platform.

### 4.5. Data Analysis of the Sequencing Results

Single-molecule real-time sequencing data were processed using SMRTlink 4.0 software. To resolve the high error rate of sub-reads, all SMRT sub-reads were corrected using the more than 300 million HiSeq reads as input data by proovread [76]. After removing the redundant sequences, all SMRT sub-reads were processed by CD-HIT-EST [77], and the full-length transcripts served as reference unigenes for subsequent analyses. Gene functions were annotated based on a series of nucleotide and protein databases, including the NCBI non-redundant protein sequence, the NCBI nucleotide sequence, the Protein Family (Pfam), euKaryotic Orthologous Groups (KOG), Swiss-Prot, KEGG, Gene Ontology (GO) databases and *Rosa chinensis* genome (assembly RchiOBHm-V2). Plant transcription factors were predicted using iTAK software [78]. All full-length transcripts were searched for the detection of SSRs by MISA (MIcroSAtellite) using default parameters. A minimum of five repetitions was considered as search criteria in the MISA script for identification of mono-to hexanucleotide motifs.

Clean data were filtered from the raw NGS sequencing data, after reads with adaptors of low quality and with unknown nucleotides (>5%) were removed. The clean data were then assembled de novo into transcripts using the Trinity platform (http://trinityrnaseq.sourceforge.net/); these transcripts were then mapped to reference unigenes via RNA-seq by expectation maximization (RSEM) [79], and the read count numbers of all genes were calculated and translated to FPKM (Fragments Per Kilobase Million) gene values [80]. The DEGs were identified by DESeq, and in addition, the uni-transcripts with a p_adj_ < 0.05 were screened [81], we also selected a set of targeted transcripts by manual screening, according to the log2(fold-change) >1 or <0.5. Then, we performed a GO enrichment analysis using Goseq [82]. The KEGG enrichment analysis was performed by KOBAS (2.0) [83].

### 4.6. qRT-PCR Validation and Expression Analysis

RNA samples were isolated from petals of both rose varieties at three different developmental stages (unopened bud, opened bud, and fully open flower). Reverse transcription was performed with a PrimeScriptTM Reverse Transcriptase Reagent Kit with a gDNA Eraser (TaKaRa, Code NO. RR047A, Shiga, Otsu, Japan). Specific primers of target genes were designed with Primer-BLAST (https://www.ncbi.nlm.nih.gov/tools/primer-blast/), and the size of qRT-PCR products ranged from 100 to 200 bps—information for each of the primer pairs can be found in Table S2. The qRT-PCR analysis was performed in triplicate using TB Green^TM^ Premix Ex Taq^TM^ II (TaKaRa, Code NO. RR820Q, Shiga, Otsu, Japan) by the StepOne Plus Real-Time PCR System (Applied Biosystems). PCR amplification was performed in a volume of 20 μL, and the program was 95 °C for 30 s, and 40 cycles of 95 °C for 5 s and 60 °C for 30 s. PCR efficiencies and C_q_ of each target gene were determined by LinRegPCR; C_q_ is the PCR cycle at which the amplification curve crosses the threshold [84]. Relative quantification of specific mRNA levels was performed using the delta–delta method (ratio = 2−ΔΔCq) for quick estimation of gene expression difference [85], and the gene expression level of *Rosa* ‘Margo Koster’ at the unopened bud stage was set as the control group. The relative quantifications of target genes were normalized according to two internal reference genes, *ACTIN* and *GAPDH*. Finally, the former was used for final relative quantification.

## Figures and Tables

**Table 1:** Summary of sequencing data from PacBio platform.

RNA-Seq	Sample	Sub-Reads Base (Gb)	Sub-Reads Number	Circular Consensus Sequence	Full Length	Flnc	Average Flnc Length (bp)
Pacbio RSII	Mixture petal of two rose varieties at blooming stage	10.55	5,383,989	463,753	370,571	365,407	2591

**Table 2:** Summary of sequencing data from Illumina platform.

RNA-seq	Sample	Repetition	Read Number	Read Bases (G)	GC (%)	Q30 (%)	Total Mapping (%)
IlluminaHiSeq 2500	Petal of *Rosa* ‘Margo Koster’ at blooming stage	Rep1	52,204,870	6.52 6.55 6.54 6.54 6.53 6.55	46.3	96.8	44,427,346 (85.10%)
		Rep2	52,393,148	6.55 6.54 6.54 6.53 6.55	46.42	97.08	45,811,502 (87.44%)
		Rep3	52,361,786	6.54	46.46	97.05	43,569,408 (83.21%)
	Petal of *Rosa* ‘White Margo Koster’ at blooming stage	Rep1	52,317,316	6.54	46.29	97.06	45,439,584 (86.85%)
		Rep2	52,252,330	6.53	46.41	97.04	45,425,380 (86.93%)
		Rep3	52,424,294	6.55	46.52	97.1	43,782,470 (83.52%)

**Table 3:** Statistics of discovered simple sequence repeats (SSRs) and their various classes in rose transcriptome.

Parameters	Counts	Motifs	No. of SSR
Total number of sequences examined	107,280	Mononucleotide	56,828
Total size of examined sequences (bp)	290,704,013	Dinucleotide	25,261
Total number of identified SSRs	96,902	Trinucleotide	13,799
Number of SSR containing sequences	45,782	Tetranucleotide	550
Number of sequences containing more than one SSR	20,252	Pentanucleotide	114
Number of SSRs present in compound formation	28,391	Hexanucleotide	350

**Table 4:** FPKM (Fragments Per Kilobase Million) of key genes involved in flavonoids and associated biosynthesis in the petals of rose varieties.

Gene Name	#ID	Expression Profile (FPKM)		Log2 (Change Fold)
		*Rosa* ‘Margo Koster’	*Rosa* ‘White Margo Koster’	
*Rm4CL*	c75759/f1p0/2016 *	35.59	72.72	0.97
*RmCHS*	c73992/f1p0/1241	79.55	37.44	−0.92
*RmF3H*	c18601/f3p3/1313 *	28.11	12.28	−0.84
*RmDFR*	c10949/f1p0/1197	44.07	11.44	−0.51
*RmFLS*	c70511/f47p0/1468	4.42	249.11	0.17
*RmLDOX*	c68764/f1p8/1370	1240.90	5.67	−0.13
*RmANR*	c64060/f1p0/1292	169.81	42.56	−0.50
*RmLAR*	c21707/f3p7/1533 *	86.88	29.34	−0.64
*RmGT*	c41272/f1p0/1762	8.53	35.75	0.48
*RmCCD*	c22761/f1p25/2265 *	11.20	5.35	−0.94

* indicates the *p*-value of DEG was less than 0.05, but their adjusted *p*-value was more than 0.05.

**Table 5:** Tentative identification and quantification of anthocyanins, procyanidins, and flavonoids in rose flower.

No.	Retention Time (min)	λmax (nm)	[M]+	Fragment Ions	Tentative Identification	*Rosa* ‘MK’ (μg/g)	*Rosa* ‘WMK’ (μg/g)	Reference	Catalogue
1	9.681	280, 520	611.161	287.055	Cyanidin 3,5 diglucoside	138.679	0.000	STD	AC
2	12.396	280, 520	595.167	271.059	Pelargonidin 3,5 diglucoside	5004.735	0.000	STD	AC
3	13.569	280, 520	625.177	301.070	Peonidin, 3,5-diglucoside	5.794	0.000	STD	AC
4	18.955	280, 520	433.112	271.059	Pelargonidin 3-glucoside	618.789	0.000	[41]	AC
5	26.356	280, 520	519.114	271.059	Pelargonidin 3-glucoside-carboxyacetyl	0.000	0.000	[41]	AC
6	11.678	280	579.149	291.086	Procyanindin B1	63.072	168.931	STD	PC
7	32.757	280	595.166	566.426	Proanthocyanidins	22,932.771	27,408.189	STD	PC
8	46.510	264, 365	287.057	153.017	Kaempferol	28.553	74.894	STD	FL
9	42.813	265, 350	637.177	287.057	Multiflorin A	12,585.927	22,779.216	[41]	FL
10	42.590	254, 370	303.049	153.017	Quercetin	1.432	11.934	STD	FL
11	33.688	265, 350	449.108	287.057	Kaempferol-3-glucoside	3275.087	6992.866	[41]	FL
12	38.683	265, 350	433.113	287.057	Kaempferol-3-rhamnopyranoside (Afzelin)	4189.292	7339.989	[41]	FL
13	37.703	265, 350	419.097	287.057	Kaempferol-3-*O*-β-glucopyranosyl-7-*O*-α-rhamnopyranoside	1905.600	1880.046	[41]	FL
14	35.872	265, 350	418.090	287.057	Kaempferol-3-*O*-α-d-Arabinoside	1409.055	2876.430	[41]	FL
15	32.689	264, 348	595.166	287.057	Kaempfeol-3-rutinoside	2701.640	3043.795	STD	FL
16	29.689	255, 353	465.102	303.049	Quercetin 3-glucoside	260.776	1623.913	STD	FL
17	28.741	254, 350	611.160	303.049	Rutin	34.425	320.854	STD	FL
18	28.883	255, 352	465.102	303.049	Hyperoside	0.000	181.090	STD	FL
19	19.050	280	291.084	139.038	(−)-Epicatechin	0.000	6.821	STD	FL
20	13.429	280	291.085	139.038	(+/−)-Catechin hydrate	282.540	1534.824	STD	FL
21	14.455	221, 260, 292	169.049	125.060	Vanillic acid	141.047	72.536	STD	FL
22	14.184	240, 325	355.102	163.039	Chlorogenic acid	1.465	0.765	STD	FL
23	14.184	243, 323	355.102	163.038	Neochlorogenic acid	14.655	7.647	STD	FL

## Data Availability

The clean paired-end sequence and clean SMRT sequencing datasets were submitted to the NCBI Sequence Read Archive (SRA) under accession number SRP132317.

## Supplementary Materials

The following are available online at https://www.mdpi.com/2223-7747/8/8/267/s1, Supplemental Figure S1: Type and number of predicted transcription factors (TFs) by iTAK. Supplemental Figure S2: Gene function classification of full-length transcripts in rose variety by GO term. Supplemental Figure S3: Gene function classification of full-length transcripts in rose variety by KOG. Supplemental Figure S4: Gene function of full-length transcripts in rose variety by KEGG database. Supplemental Figure S5: Raw Cq data of qRT-PCR. Supplemental Table S1: Full-length transcripts of isoforms involved in pigment biosynthesis in rose flowers. Supplemental Table S2: Primer sequences information used in qRT-PCR. Supplemental Table S3: PCR efficiency of each mRNA from preliminary qRT-PCR. Supplemental File S1: List of DEGs between *Rosa* ‘Margo Koster’ and *Rosa* ‘White Margo Koster’. Supplemental File S2: Qualification and annotation of all expressed genes in two libraries. Supplemental File S3: Primers information of candidates SSR markers.
