# Supplementary material for: Hybrid-Transcriptome Sequencing and Associated Metabolite Analysis Reveal Putative Genes Involved in Flower Color Difference in Rose Mutants
Source: Plants (Basel). 2019 Aug 5;8(8):267. doi: 10.3390/plants8080267 (PMC6724100; doi:10.3390/plants8080267)
Supplement: Supplementary file 1 [file plants-08-00267-s001.zip › Suppl. Figure 5. Raw Cq data of qRT-PCR.docx]

**Suppl. Figure 5.** Raw Cq data of qRT-PCR
