# Supplementary material for: Hybrid-Transcriptome Sequencing and Associated Metabolite Analysis Reveal Putative Genes Involved in Flower Color Difference in Rose Mutants
Source: Plants (Basel). 2019 Aug 5;8(8):267. doi: 10.3390/plants8080267 (PMC6724100; doi:10.3390/plants8080267)
Supplement: Supplementary file 1 [file plants-08-00267-s001.zip › Suppl. Table 1. Full-length transcripts of isoforms involved in pigment biosynthesis in rose flowers.docx]

**Suppl. Table 1.** Full-length transcripts of isoforms involved in pigment biosynthesis in rose flowers

| **KEGG Name.** | **KEGG No.** | **Gene annotation** | **Transcripts No.** | **Total** |
| --- | --- | --- | --- | --- |
| Flavonoid biosynthesis | ko00941 | shikimate O-hydroxycinnamoyltransferase | 12 | 197 |
|  |  | anthocyanidin reductase | 15 |  |
|  |  | bifunctional dihydroflavonol 4-reductase/flavanone 4-reductase | 10 |  |
|  |  | leucoanthocyanidin reductase | 2 |  |
|  |  | chalcone isomerase | 3 |  |
|  |  | naringenin 3-dioxygenase | 15 |  |
|  |  | leucoanthocyanidin dioxygenase | 30 |  |
|  |  | chalcone synthase | 62 |  |
|  |  | coumaroylquinate 3'-monooxygenase | 2 |  |
|  |  | caffeoyl-CoA O-methyltransferase | 14 |  |
|  |  | flavonol synthase | 19 |  |
|  |  | trans-cinnamate 4-monooxygenase | 7 |  |
|  |  | flavonoid 3'-monooxygenase | 6 |  |
| Flavone and flavonol biosynthesis | ko00944 | flavonol 3-O-glucosyltransferase | 1 | 16 |
|  |  | UDP-glucosyl transferase 73C | 9 |  |
|  |  | flavonoid 3'-monooxygenase | 6 |  |
| Anthocyanin biosynthesis | ko00942 | anthocyanidin 5,3-O-glucosyltransferase | 7 | 7 |
