# Supplementary material for: Hybrid-Transcriptome Sequencing and Associated Metabolite Analysis Reveal Putative Genes Involved in Flower Color Difference in Rose Mutants
Source: Plants (Basel). 2019 Aug 5;8(8):267. doi: 10.3390/plants8080267 (PMC6724100; doi:10.3390/plants8080267)
Supplement: Supplementary file 1 [file plants-08-00267-s001.zip › Suppl. Table 2. Primer sequences information used in qRT-PCR.docx]

**Suppl. Table 2.** Primer sequences information used in qRT-PCR

| **Gene name** | **Predict gene accession** | **Forward primer (5’-3’)** | **Reserve primer(5’-3’)** | **Product size (bps)** |
| --- | --- | --- | --- | --- |
| *CHS1* | AEC13058.1 | AGACCGTCGTGCTTCACAG | GATGCCCACACACGCCTTTA | 167 |
| *CHS2* | AB038246.1 | ACTTGGCTAATCTGCTCCTGG | GTTTACCCTCAGAATGCCCAA | 133 |
| *CHI* | XM_024321061.1 | TGAAGCAAGGAAATGTGTGGC | TCTCCTTTCTCAGTTTCATGCCT | 121 |
| *F3H* | XM_024316694 | ACGCATTTGATTGGGCCTTG | TCAAACCGGCCACACTAGAA | 185 |
| *FLS* | ABH07784.1 | TTACTGCAGGGGGTGTCTCA | CACAAACTCTCAGTTTCACGCC | 124 |
| *DFR* | D85102.1 | ATCGCACGATGCTACGATTCA | ATGCCCTTGAACCTGCACT | 168 |
| *LDOX* | XP_004298720.1 | GAGCTGCTCATATCCCCACT | AGTGATCTCCCACAACATCGTC | 149 |
| *GT* | AB201049.1 | AAGTTGGCTCAGCTGTGGA | CGGCACAAACATGCCATTAC | 192 |
| *UFGT* | BAK09602.1 | TCAAGAGGCTGTAGGACCCA | CTTTCACCCACCTGAGGACC | 150 |
| *ANR* | XP_004306690.1 | GCTACCAAGCTTAGTGCGGA | GCTCAAACGTAACAAGGCAGA | 131 |
| *LAR* | XM_024340977.1 | GGACCTTGGATGAGTGCTTCA | AACTCTTGGGAGCATCAGGG | 164 |
| *ACTIN* | XM_024323957.1 | GTCTCGGTTGTGCTCCATCT | GTTGGTGCATGGTCTCGGTA | 195 |
| *GAPDH* | XM_024328179.1 | TGGTGATGGGAGATGACATGG | CGCTAGCTAGTTCGACAGACC | 151 |
| *MYBPA* | NP_001295449.1 | GGCCCTTCTGAAAACGACAGA | TTGCGCGCGAGAGAGAGATAA | 137 |
| *MYBAN2* | AID23892.1 | GCCCAAGCTTCAAAATACGC | GCCACTGATGCTTTGCTACA | 110 |
