# Supplementary material for: Hybrid-Transcriptome Sequencing and Associated Metabolite Analysis Reveal Putative Genes Involved in Flower Color Difference in Rose Mutants
Source: Plants (Basel). 2019 Aug 5;8(8):267. doi: 10.3390/plants8080267 (PMC6724100; doi:10.3390/plants8080267)
Supplement: Supplementary file 1 [file plants-08-00267-s001.zip › Suppl. Table 3. PCR efficiency of each mRNA from preliminary qPCR.docx]

**Suppl. Table 3.** PCR efficiency of each mRNA from preliminary qPCR

| **mRNA** | **Predict gene accession** | **Mean Efficiency** | **St Dev** | **SEM** | **RSE** |
| --- | --- | --- | --- | --- | --- |
| *CHS1* | AEC13058.1 | 1.880 | 0.009 | 0.002 | 0.001 |
| *CHS2* | AB038246.1 | 1.907 | 0.008 | 0.002 | 0.001 |
| *CHI* | XM_024321061.1 | 1.888 | 0.014 | 0.003 | 0.002 |
| *F3H* | XM_024316694 | 1.877 | 0.017 | 0.003 | 0.002 |
| *FLS* | ABH07784.1 | 1.924 | 0.012 | 0.003 | 0.001 |
| *DFR* | D85102.1 | 1.905 | 0.011 | 0.002 | 0.001 |
| *LDOX* | XP_004298720.1 | 1.892 | 0.013 | 0.003 | 0.001 |
| *GT* | AB201049.1 | 1.876 | 0.017 | 0.004 | 0.002 |
| *UFGT* | BAK09602.1 | 1.868 | 0.009 | 0.002 | 0.001 |
| *ANR* | XP_004306690.1 | 1.885 | 0.010 | 0.002 | 0.001 |
| *LAR* | XM_024340977.1 | 1.889 | 0.019 | 0.004 | 0.002 |
| *MYBPA* | NP_001295449.1 | 1.918 | 0.014 | 0.003 | 0.002 |
| *ACT* | XM_024323957.1 | 1.896 | 0.015 | 0.003 | 0.002 |
| *GAPDH* | XM_024328179.1 | 1.896 | 0.011 | 0.004 | 0.002 |
| *MYBAN2* | AID23892.1 | 1.904 | 0.013 | 0.003 | 0.001 |

R^2^ of linear regression for each mRNA was greater than 0.995
