# Supplementary material for: Hybrid-Transcriptome Sequencing and Associated Metabolite Analysis Reveal Putative Genes Involved in Flower Color Difference in Rose Mutants
Source: Plants (Basel). 2019 Aug 5;8(8):267. doi: 10.3390/plants8080267 (PMC6724100; doi:10.3390/plants8080267)
Supplement: Supplementary file 1 [file plants-08-00267-s001.zip › supplyment data list.docx]

**Supplementary Materials:** The following are available online at [www.mdpi.com/xxx/s1](http://www.mdpi.com/xxx/s1)

Suppl. Figure 1. Type and number of predicted transcription factors (TFs) by iTAK.

Suppl. Figure 2. Gene function classification of full-length transcripts in rose variety by GO term

Suppl. Figure 3. Gene function classification of full-length transcripts in rose variety by KOG

Suppl. Figure 4. Gene function of full-length transcripts in rose variety by KEGG database

Suppl. Figure 5. Raw Cq data of qRT-PCR

Suppl. Table 1. Full-length transcripts of isoforms involved in pigment biosynthesis in rose flowers

Suppl. Table 2. Primer sequences information used in qRT-PCR

Suppl. Table 3. PCR efficiency of each mRNA from preliminary qPCR

Suppl. File 1. Qualification and annotation of all expressed genes in two libraries.

Suppl. File 2. List of DEGs between *Rosa* ‘Margo Koster’ and *Rosa* ‘White Margo Koster’.

Suppl. File 3. Primers information of candidates SSR markers.

**
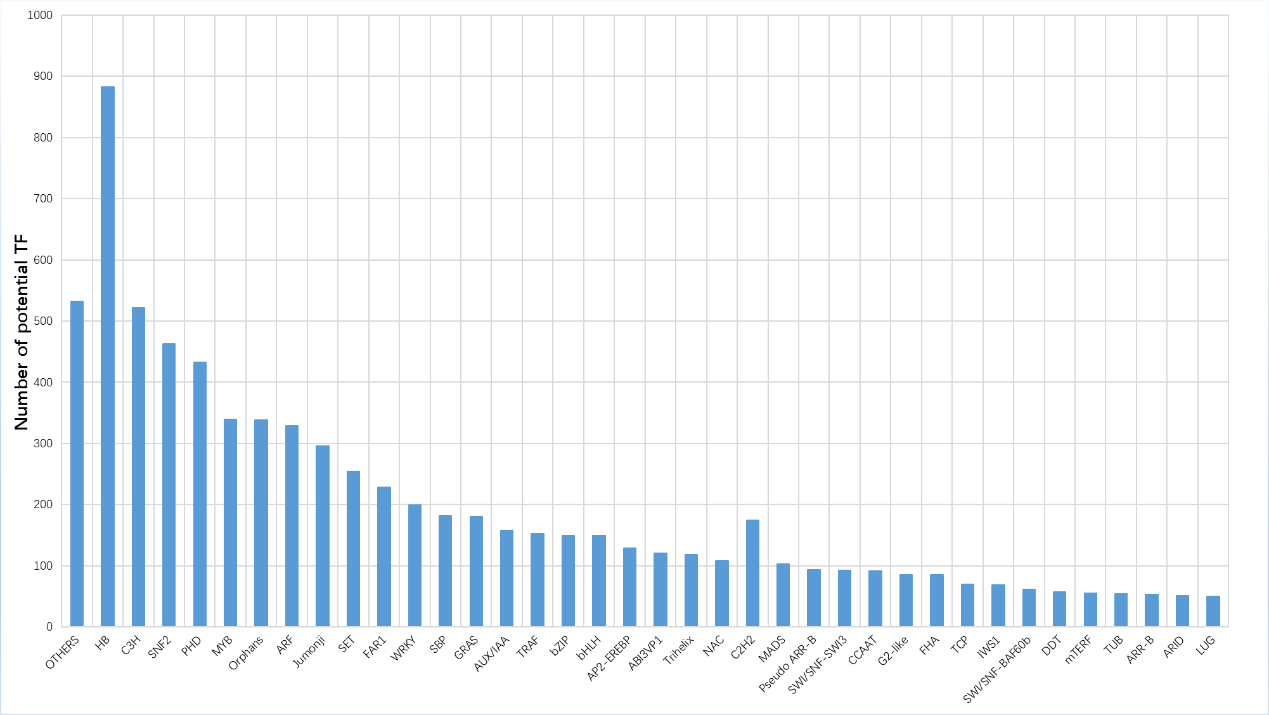
**

**Suppl. Figure 1.** Type and number of predicted transcription factors (TFs) by iTAK.


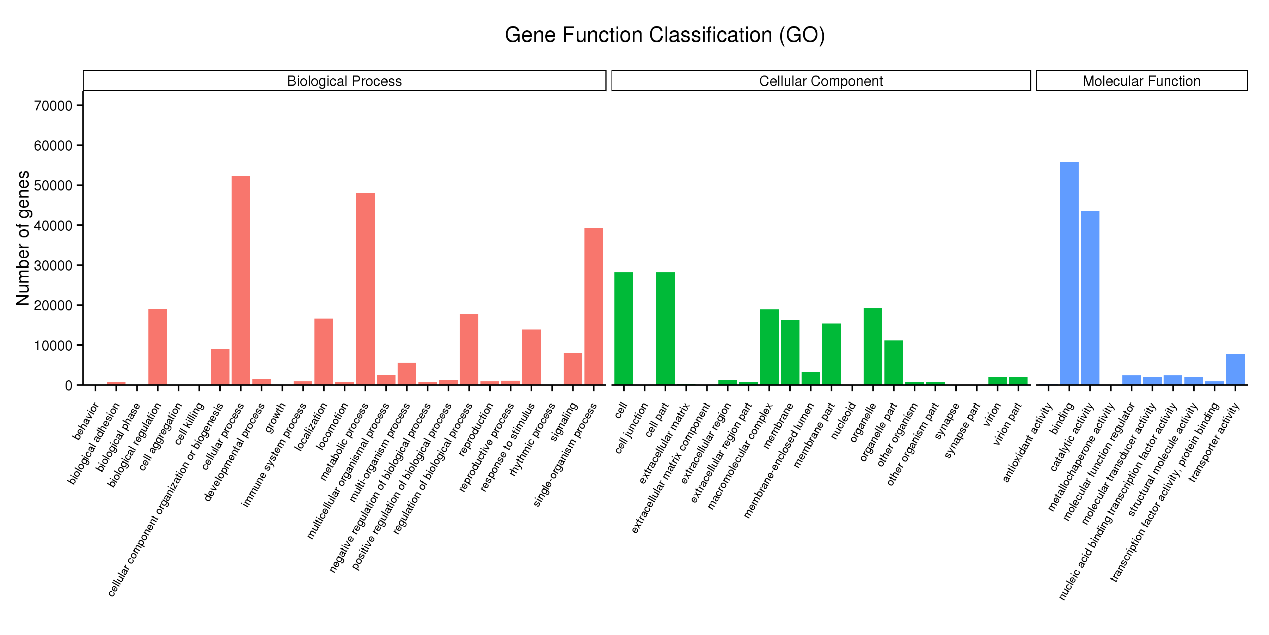


**Suppl. Figure 2.** Gene function classification of full-length transcripts in rose variety by GO term


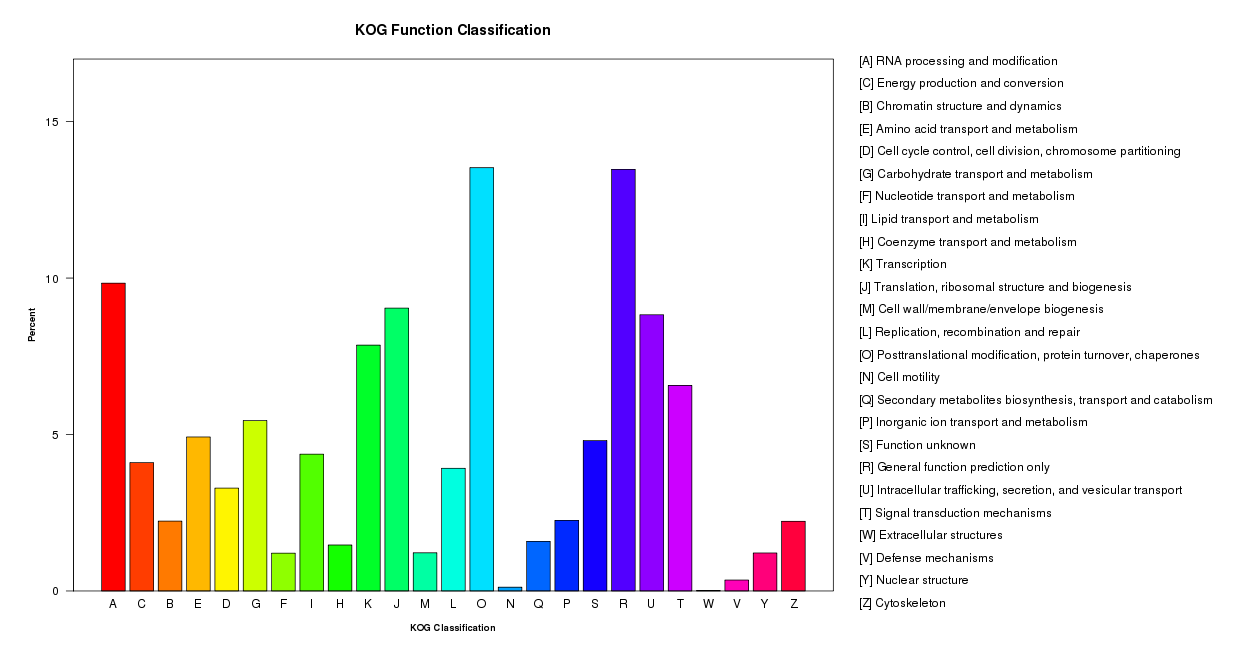


**Suppl. Figure 3.** Gene function classification of full-length transcripts in rose variety by KOG


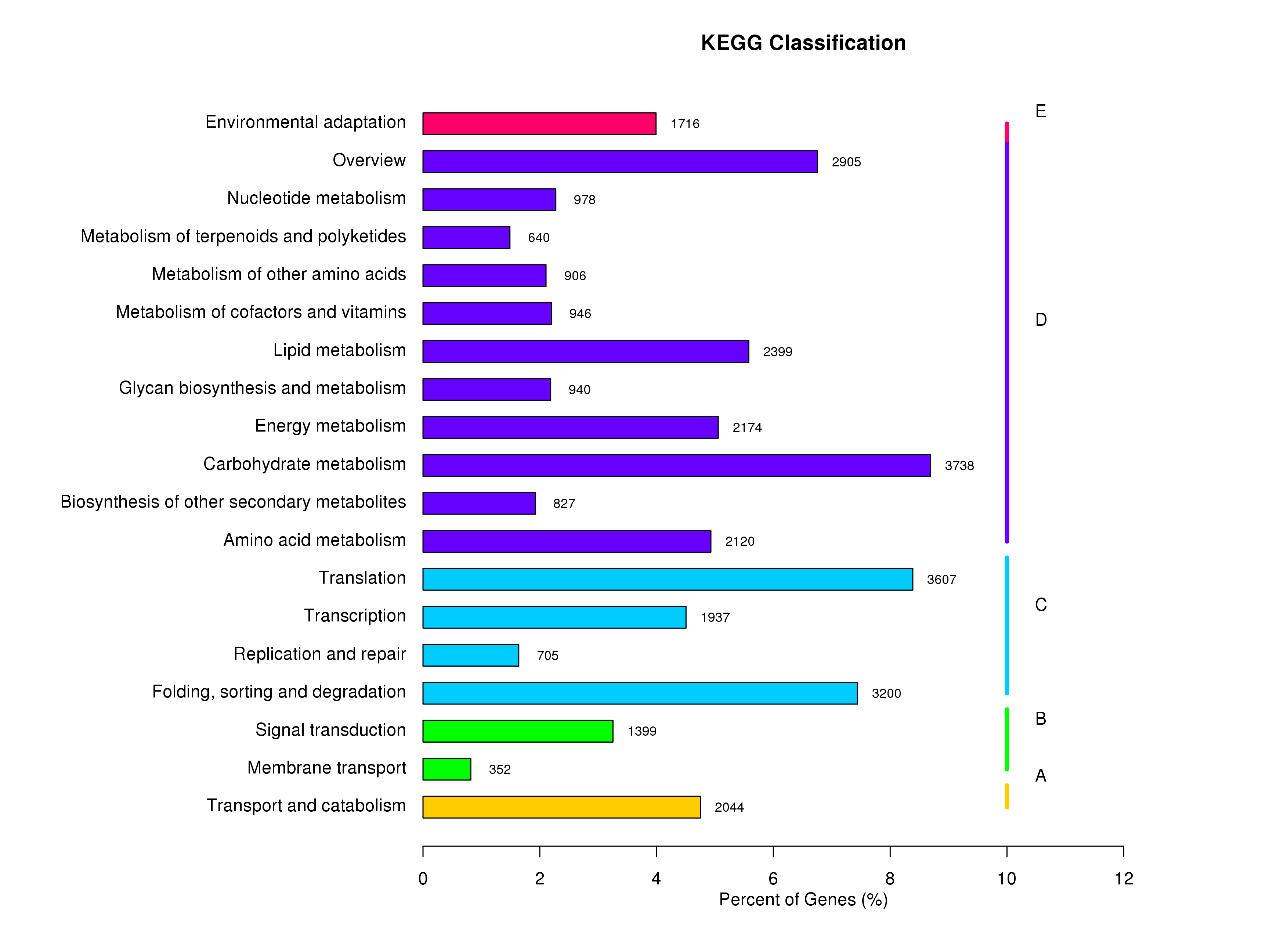


**Suppl. Figure 4.** Gene function of full-length transcripts in rose variety by KEGG database

**Suppl. Figure 5.** Raw Cq data of qRT-PCR

**Suppl. Table 1.** Full-length transcripts of isoforms involved in pigment biosynthesis in rose flowers

| **KEGG Name.** | **KEGG No.** | **Gene annotation** | **Transcripts No.** | **Total** |
| --- | --- | --- | --- | --- |
| Flavonoid biosynthesis | ko00941 | shikimate O-hydroxycinnamoyltransferase | 12 | 197 |
|  |  | anthocyanidin reductase | 15 |  |
|  |  | bifunctional dihydroflavonol 4-reductase/flavanone 4-reductase | 10 |  |
|  |  | leucoanthocyanidin reductase | 2 |  |
|  |  | chalcone isomerase | 3 |  |
|  |  | naringenin 3-dioxygenase | 15 |  |
|  |  | leucoanthocyanidin dioxygenase | 30 |  |
|  |  | chalcone synthase | 62 |  |
|  |  | coumaroylquinate 3'-monooxygenase | 2 |  |
|  |  | caffeoyl-CoA O-methyltransferase | 14 |  |
|  |  | flavonol synthase | 19 |  |
|  |  | trans-cinnamate 4-monooxygenase | 7 |  |
|  |  | flavonoid 3'-monooxygenase | 6 |  |
| Flavone and flavonol biosynthesis | ko00944 | flavonol 3-O-glucosyltransferase | 1 | 16 |
|  |  | UDP-glucosyl transferase 73C | 9 |  |
|  |  | flavonoid 3'-monooxygenase | 6 |  |
| Anthocyanin biosynthesis | ko00942 | anthocyanidin 5,3-O-glucosyltransferase | 7 | 7 |

**Suppl. Table 2.** Primer sequences information used in qRT-PCR

| **Gene name** | **Predict gene accession** | **Forward primer (5’-3’)** | **Reserve primer(5’-3’)** | **Product size (bps)** |
| --- | --- | --- | --- | --- |
| *CHS1* | AEC13058.1 | AGACCGTCGTGCTTCACAG | GATGCCCACACACGCCTTTA | 167 |
| *CHS2* | AB038246.1 | ACTTGGCTAATCTGCTCCTGG | GTTTACCCTCAGAATGCCCAA | 133 |
| *CHI* | XM_024321061.1 | TGAAGCAAGGAAATGTGTGGC | TCTCCTTTCTCAGTTTCATGCCT | 121 |
| *F3H* | XM_024316694 | ACGCATTTGATTGGGCCTTG | TCAAACCGGCCACACTAGAA | 185 |
| *FLS* | ABH07784.1 | TTACTGCAGGGGGTGTCTCA | CACAAACTCTCAGTTTCACGCC | 124 |
| *DFR* | D85102.1 | ATCGCACGATGCTACGATTCA | ATGCCCTTGAACCTGCACT | 168 |
| *LDOX* | XP_004298720.1 | GAGCTGCTCATATCCCCACT | AGTGATCTCCCACAACATCGTC | 149 |
| *GT* | AB201049.1 | AAGTTGGCTCAGCTGTGGA | CGGCACAAACATGCCATTAC | 192 |
| *UFGT* | BAK09602.1 | TCAAGAGGCTGTAGGACCCA | CTTTCACCCACCTGAGGACC | 150 |
| *ANR* | XP_004306690.1 | GCTACCAAGCTTAGTGCGGA | GCTCAAACGTAACAAGGCAGA | 131 |
| *LAR* | XM_024340977.1 | GGACCTTGGATGAGTGCTTCA | AACTCTTGGGAGCATCAGGG | 164 |
| *ACTIN* | XM_024323957.1 | GTCTCGGTTGTGCTCCATCT | GTTGGTGCATGGTCTCGGTA | 195 |
| *GAPDH* | XM_024328179.1 | TGGTGATGGGAGATGACATGG | CGCTAGCTAGTTCGACAGACC | 151 |
| *MYBPA* | NP_001295449.1 | GGCCCTTCTGAAAACGACAGA | TTGCGCGCGAGAGAGAGATAA | 137 |
| *MYBAN2* | AID23892.1 | GCCCAAGCTTCAAAATACGC | GCCACTGATGCTTTGCTACA | 110 |

**Suppl. Table 3.** PCR efficiency of each mRNA from preliminary qPCR

| **mRNA** | **Predict gene accession** | **Mean Efficiency** | **St Dev** | **SEM** | **RSE** |
| --- | --- | --- | --- | --- | --- |
| *CHS1* | AEC13058.1 | 1.880 | 0.009 | 0.002 | 0.001 |
| *CHS2* | AB038246.1 | 1.907 | 0.008 | 0.002 | 0.001 |
| *CHI* | XM_024321061.1 | 1.888 | 0.014 | 0.003 | 0.002 |
| *F3H* | XM_024316694 | 1.877 | 0.017 | 0.003 | 0.002 |
| *FLS* | ABH07784.1 | 1.924 | 0.012 | 0.003 | 0.001 |
| *DFR* | D85102.1 | 1.905 | 0.011 | 0.002 | 0.001 |
| *LDOX* | XP_004298720.1 | 1.892 | 0.013 | 0.003 | 0.001 |
| *GT* | AB201049.1 | 1.876 | 0.017 | 0.004 | 0.002 |
| *UFGT* | BAK09602.1 | 1.868 | 0.009 | 0.002 | 0.001 |
| *ANR* | XP_004306690.1 | 1.885 | 0.010 | 0.002 | 0.001 |
| *LAR* | XM_024340977.1 | 1.889 | 0.019 | 0.004 | 0.002 |
| *MYBPA* | NP_001295449.1 | 1.918 | 0.014 | 0.003 | 0.002 |
| *ACT* | XM_024323957.1 | 1.896 | 0.015 | 0.003 | 0.002 |
| *GAPDH* | XM_024328179.1 | 1.896 | 0.011 | 0.004 | 0.002 |
| *MYBAN2* | AID23892.1 | 1.904 | 0.013 | 0.003 | 0.001 |

R2 of linear regression for each mRNA was greater than 0.995
