# Supplementary figures and images for: Hybrid-Transcriptome Sequencing and Associated Metabolite Analysis Reveal Putative Genes Involved in Flower Color Difference in Rose Mutants
Source: Plants (Basel). 2019 Aug 5;8(8):267. doi: 10.3390/plants8080267 (PMC6724100; doi:10.3390/plants8080267)

**
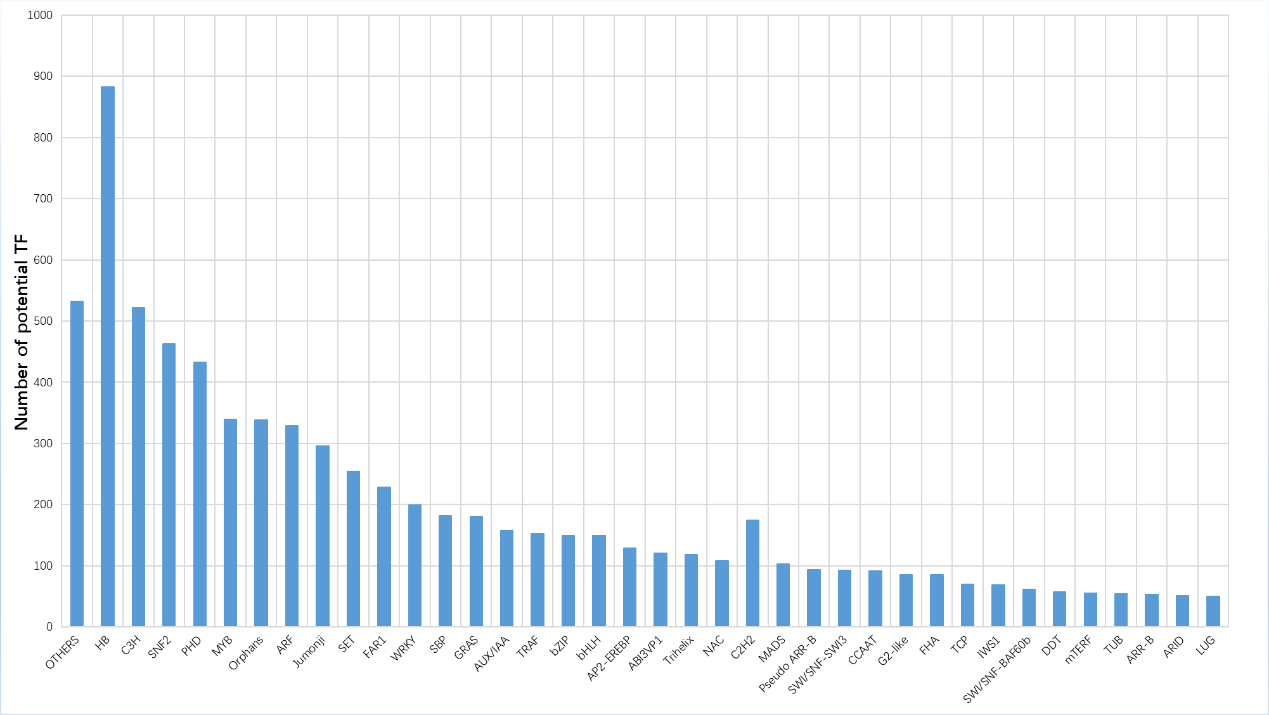
**

**Suppl. Figure 1.** Type and number of predicted transcription factors (TFs) by iTAK.

Supplement: Supplementary file 1 [file plants-08-00267-s001.zip › Suppl. Figure 1. Type and number of predicted transcription factors (TFs) by iTAK..docx]

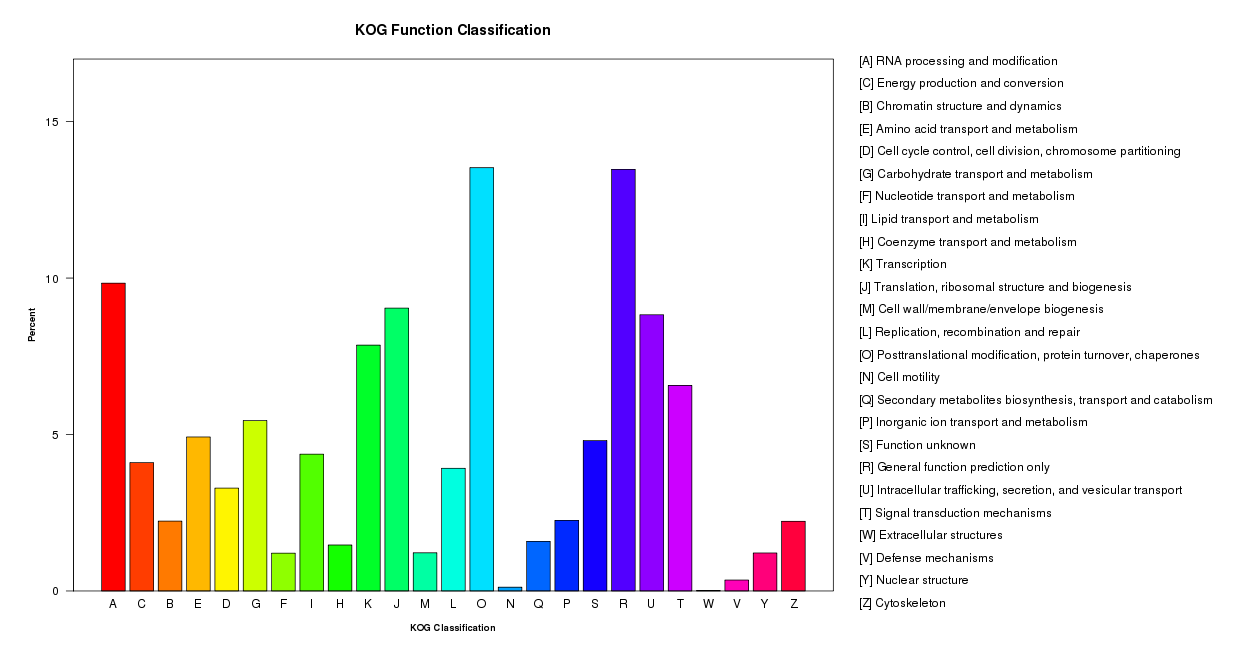


**Suppl. Figure 3.** Gene function classification of full-length transcripts in rose variety by KOG

Supplement: Supplementary file 1 [file plants-08-00267-s001.zip › Suppl. Figure 3. Gene function classification of full-length transcripts in rose variety by KOG.docx]

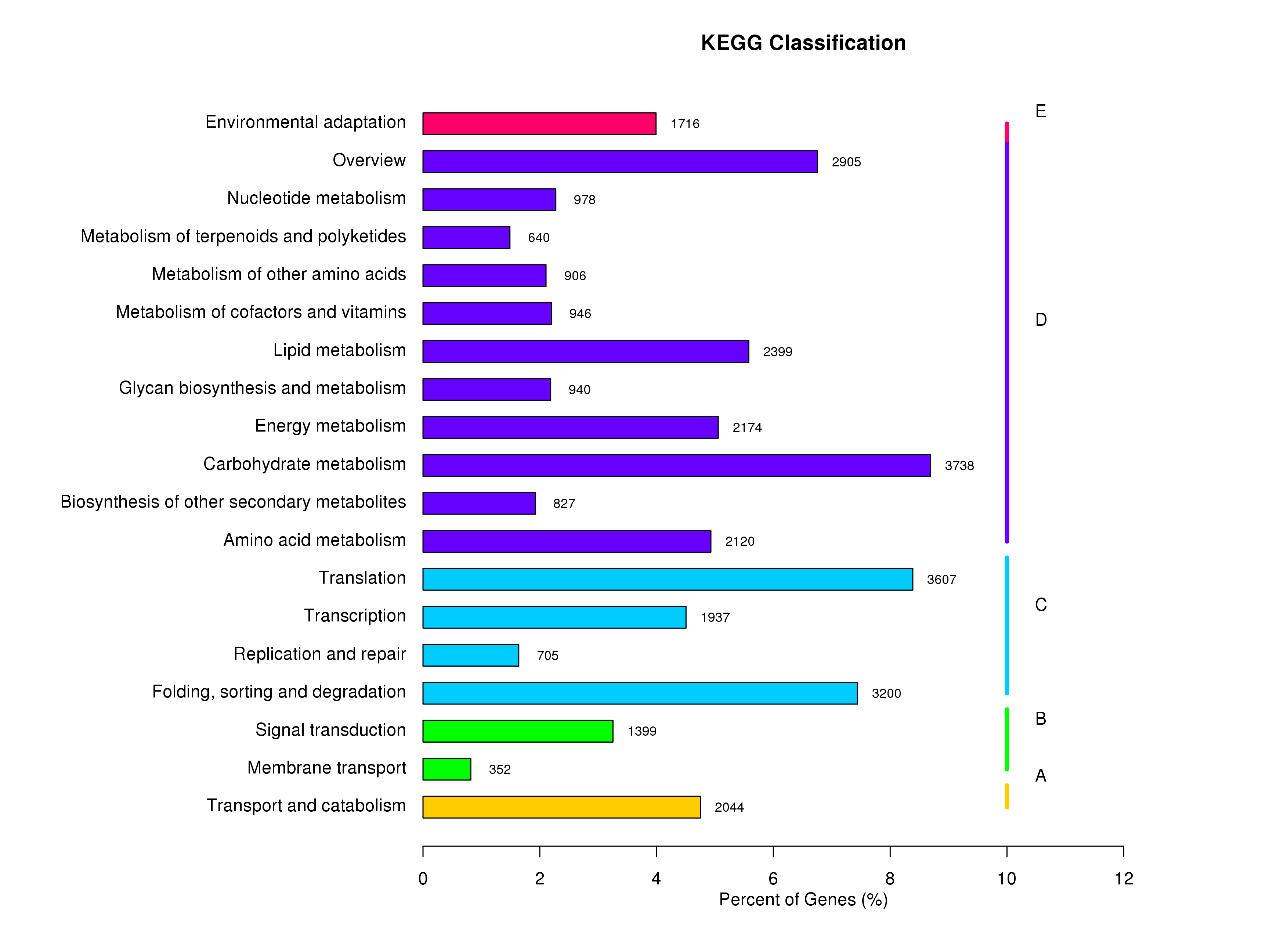


**Suppl. Figure 4.** Gene function of full-length transcripts in rose variety by KEGG database

Supplement: Supplementary file 1 [file plants-08-00267-s001.zip › Suppl. Figure 4. Gene function of full-length transcripts in rose variety by KEGG database.docx]
